# Transcriptomic Analysis of Monocyte-Derived Non-Phagocytic Macrophages Favors a Role in Limiting Tissue Repair and Fibrosis

**DOI:** 10.3389/fimmu.2020.00405

**Published:** 2020-03-31

**Authors:** Sergei Butenko, Senthil K. Satyanarayanan, Simaan Assi, Sagie Schif-Zuck, Dalit Barkan, Noa Sher, Amiram Ariel

**Affiliations:** ^1^Department of Human Biology, University of Haifa, Haifa, Israel; ^2^Tauber bioinformatics Center, University of Haifa, Haifa, Israel

**Keywords:** inflammation, macrophages, efferocytosis, transcriptional profiling, fibrosis

## Abstract

Monocyte-derived macrophages are readily differentiating cells that adapt their gene expression profile to environmental cues and functional needs. During the resolution of inflammation, monocytes initially differentiate to reparative phagocytic macrophages and later to pro-resolving non-phagocytic macrophages that produce high levels of IFNβ to boost resolutive events. Here, we performed in-depth analysis of phagocytic and non-phagocytic myeloid cells to reveal their distinct features. Unexpectedly, our analysis revealed that the non-phagocytic compartment of resolution phase myeloid cells is composed of Ly6C^med^F4/80^−^ and Ly6C^hi^F4/80^lo^ monocytic cells in addition to the previously described Ly6C^−^F4/80^+^ satiated macrophages. In addition, we found that both Ly6C^+^ monocytic cells differentiate to Ly6C^−^F4/80^+^macrophages, and their migration to the peritoneum is CCR2 dependent. Notably, satiated macrophages expressed high levels of IFNβ, whereas non-phagocytic monocytes of either phenotype did not. A transcriptomic comparison of phagocytic and non-phagocytic resolution phase F4/80^+^ macrophages showed that both subtypes express similar gene signatures that make them distinct from other myeloid cells. Moreover, we confirmed that these macrophages express closer transcriptomes to monocytes than to resident peritoneal macrophages (RPM) and resemble resolutive Ly6C^lo^ macrophages and monocyte-derived macrophages more than their precursors, inflammatory Ly6C^hi^ monocytes, recovered following liver injury and healing, and thioglycolate-induced peritonitis, respectively. A direct comparison of these subsets indicated that the non-phagocytic transcriptome is dominated by satiated macrophages and downregulate gene clusters associated with excessive tissue repair and fibrosis, ROS and NO synthesis, glycolysis, and blood vessel morphogenesis. On the other hand, non-phagocytic macrophages enhance the expression of genes associated with migration, oxidative phosphorylation, and mitochondrial fission as well as anti-viral responses when compared to phagocytic macrophages. Notably, conversion from phagocytic to satiated macrophages is associated with a reduction in the expression of extracellular matrix constituents that were demonstrated to be associated with idiopathic pulmonary fibrosis (IPF). Thus, macrophage satiation during the resolution of inflammation seems to bring about a transcriptomic transition that resists tissue fibrosis and oxidative damage while promoting the restoration of tissue homeostasis to complete the resolution of inflammation.

## Introduction

Acute inflammation is the protective response of the host to damaging events that may interrupt tissue homeostasis, such as physical or chemical injury, as well as microbial infections. A successful response eliminates the threat locally, repair the affected tissue, and restore its structure and function without deleterious fibrosis. Inflammation initiates with the production of soluble mediators by resident cells in the injured/infected tissue that promote the exudation of defense and/or signaling proteins, reinforced by the influx of granulocytes from the blood. Upon the arrival of these leukocytes, mostly neutrophils, they primarily function to phagocytose and eliminate foreign microorganisms via distinct intracellular killing mechanisms, resulting in neutrophils undergoing programmed cell death (apoptosis) (1, 2). This occurs alongside monocyte influx and their maturation into inflammatory macrophages upon infiltration of the inflamed tissue. Macrophages engulf apoptotic polymorphonuclear neutrophils (PMN) in a nonphlogistic process termed efferocytosis (1, 3). This clearance process initiates an active anti-inflammatory and pro-resolution phase that blocks excessive neutrophil recruitment and eliminates the early inflammatory elements and, in turn, results in clearance of these macrophages by either *in situ* apoptosis or egression via the lymphatic system (4, 5). Inflammatory macrophages polarize to distinct subpopulations following exposure to different bioactive molecules and environments. These subpopulations compose a wide spectrum of phenotypes that range from classically (M1) to alternatively (M2) activated macrophages—two commonly used myeloid measuring sticks generated during responses to bacterial or helminth infections and support Th1 or Th2 development, respectively (6). Recent molecular studies indicate that macrophage differentiation at different tissues and activation under different settings is associated with substantial shifts in gene expression patterns (hundreds of genes) depending on the specific stimuli (7–9). Nevertheless, most of these patterns define a distinct activation state of macrophages that cannot be confined to an M1 or M2 phenotype. As a result, the current literature promotes the usage of marker combinations or inducing agents to ascribe macrophage phenotypes rather than the M1 and M2 extremes (6, 10, 11).

Engulfment of apoptotic cells evokes signaling events that block the release of pro-inflammatory mediators from macrophages stimulated by microbial moieties, a phenomenon termed immune silencing. This process is accompanied by the production of cytokines that can promote the resolution of inflammation and wound repair (e.g., TGFβ and IL-10) (12, 13) with the production of pro-resolving lipid mediators, such as resolvin (Rv) E1 and RvD1 that block PMN infiltration and promote their clearance (1). Notably, the uptake and processing of high amounts of biopolymers, such as the ones expressed by apoptotic cells, require coping with large amounts of reactive oxygen species (ROS) generated in metabolic processes. Therefore, mitochondrial ROS production is limited in high-burden efferocytic macrophages, by means such as mitochondrial fission and lowering mitochondrial membrane potential (14, 15), to allow continued engulfment and avoid oxidative damage. Recent studies in spontaneously resolving, zymosan A-induced murine peritonitis characterized macrophages from resolving peritonitis into two distinct subtypes based on differing surface expression of the adhesion molecule CD11b that also composes complement receptor 3 (CR3) that mediates apoptotic cell engulfment by human macrophages (16). Compared to their CD11b^high^ counterparts, the CD11b^low^ macrophages are characterized by lower levels of pro-inflammatory mediators (e.g., TNFα, IL-1β, CCL2, 3, and 5) and proteins (e.g., iNOS and COX2), and pro-fibrotic factors (e.g., arginase-1). However, they display a higher secretion of the anti-infammatory/pro-resolving cytokine TGFβ and higher expression of the pro-resolving enzyme 12/15-lipoxygenase (LO). CD11b^low^ macrophages migrate out of inflamed sites and, compared to CD11b^high^ cells, exert decreased phagocytic activity despite containing higher numbers of PMNs previously engulfed. Hence, they were termed satiated or non-phagocytic macrophages (17, 18). A similar series of phenotypic transitions by monocyte-derived macrophages was previously reported in acute liver injury, where Ly6C^hi^ monocytes infiltrate the liver, clear apoptotic neutrophils, and convert to Ly6C^lo^ macrophages that express 12/15-LO (19–21).

Recently, IFNβ expression by non-phagocytic macrophages, and the novel roles of this cytokine as an effector in resolving bacterial inflammation were reported (17). We aimed to identify the satiated macrophage subset within the non-phagocytic macrophage population, determine the transcriptomic origin of resolution phase macrophages (of both the phagocytic and non-phagocytic phenotypes), and identify the unique gene clusters expressed by non-phagocytic/satiated macrophages. Furthermore, we sought to determine whether these unique clusters support key effector functions of satiated macrophages. Such functions include loss of phagocytic/efferocytic capacity while maintaining low ROS burden, deviation from the M2-like/reparative/pro-fibrotic phenotype to a pro-resolving phenotype, and metabolic shifts between various metabolic pathways. Here, we report that resolution phase non-phagocytic myeloid cells are composed of two distinct subsets, in addition to satiated macrophages. However, the Ly6C^+^ subsets are not becoming phagocytic prior to differentiation and do not express high levels of IFNβ as the satiated macrophages. We also found that both phagocytic and non-phagocytic resolution phase macrophages express a transcriptome that is more similar to the one expressed by monocytes than to RPM and more similar to reparative Ly6C^lo^ monocyte-derived macrophages than inflammatory Ly6C^hi^ monocytes from liver injury or peritoneal thioglycolate challenge. In addition, we found non-phagocytic macrophages to display a satiation-associated transcriptome with a significant change in expression patterns between phagocytic and satiated macrophages that attest to a complete phenotype switch in satiated macrophages that involves phagocytic properties, tissue repair and fibrosis, and metabolic programs.

## Materials and Methods

### Mice

C57BL/6 WT male mice were purchased from Harlan Laboratories. All mice that were used at the age of 8–15 weeks and did not undergo previous procedures. All mice were housed under a 12-h:12-h light–dark cycle and specific pathogen-free conditions, up to five mice per cage. Mice were fed standard pellet chow and reverse osmosis water *ad libitum*. Animal experiments were approved by the Committee of Ethics, University of Haifa (authorization no. 246/14).

### Murine Peritonitis

Male C57BL/6 mice were randomly assigned to experimental groups. Mice were injected I.P. with zymosan A (1 mg/ml in PBS, 1 ml per mouse). PKH2-PCL green (0.25 mM; 0.5 ml; Sigma-Aldrich) was injected I.P. at 20, 44, 62, or 68 h, and peritoneal exudates were collected 4 h later. Peritoneal cells were stained with PE- or Brilliant violet-conjugated rat anti-mouse F4/80, PerCP-conjugated rat anti-mouse CD11b, Pacific Blue- or PerCP-conjugated rat anti-mouse Ly6C, PE-conjugated rat anti-mouse CD115, and PE/Cy7-conjugated mouse anti-mouse CX_3_CR1 (Biolegend) and analyzed by flow cytometry as in Supplemental Figure 1. F4/80^+^macrophages were sorted according to PKH2-PCL green signal intensity as in (17) using the FACSaria III sorter (Beckton-Dickinson) to give distinct F4/80^+^/PKH2^hi^ and F4/80^+^/PKH2^lo/neg^ macrophage populations. Ly6C^med^F4/80^neg^ and Ly6C^hi^F4/80^lo^ monocytic cells were sorted using the SH800 sorter (Sony). In some experiments, flow cytometry analysis using the FlowJo software (Treestar) was performed to identify distinct leukocyte populations as detailed in the results section.

### Monocytic Ablation

To ablate monocyte migration to the peritoneum, mice received 400 μl of anti-mouse CCR2 mAb (clone MC-21, generously given by Prof. Mack, Regensburg, Germany) conditioned media (29 μg Ab/ml, I.P.) concomitantly with zymosan A peritonitis onset (0 h) and at 24 h PPI.

### RNA Isolation

RNA extraction was performed as previously described (17). Briefly, all RNA species from sorted cells were extracted using the Aurum Total RNA kit (Bio-Rad Laboratories, Inc.). RNA integrity was scored by Agilent 2100 Bioanalyzer using the Agilent RNA 6000 Pico kit (Agilent Technologies). Samples were prepared for Illumina sequencing using NEB's Ultra Directional RNA Library Prep Kit for Illumina (NEB#7420). Libraries were sequenced with a 50 bp SR run on Illumina HiSeq 2500 using a V3 flow cell.

### Data Processing and Analysis

Sequenced reads were compared to available murine Ensembl 70 genes using mouse genome build (GRCm38), and expression was compared between PKH2^hi^ and PKH2^lo^ macrophages using two separate analysis pipelines: RSEM/EdgeR and TopHat2/cuffdiff. Depending on the pipeline, between ~3,300 and 3,400 genes were found to be differentially expressed (FDR ≤ 0.05), with a wide overlap in results between the two pipelines. Significance values presented were from the TopHat2/cuffdiff analysis. Differentially expressed genes with statistical significance were filtered and visualized through a volcano plot, where F4/80^+^/PKH2^hi^ cells served as a reference sample. Genes with FDR adjusted *p*-value (*q* value) ≤ 0.05 were considered as genes exhibiting differential expression between the two macrophage subsets and were selected for enriched gene ontology (GO) analysis. GO enrichment analysis was performed on the differentially up- or downregulated genes with the DAVID Bioinformatics Resources 6.7 software using the annotation categories of GOTERM_BP_5 and KEGG_PATHWAY, similarity threshold 0.7, and EASE score 0.25. For HeatMap analyses, expression values of genes were rescaled to a mean of 0 and a standard deviation of 1, and hierarchical clustering was performed using the R package Superheat with Euclidean distance and complete linkage methods (22). Published datasets were obtained in the form of gene raw counts or CPM-TMM normalized values at GREIN (23). For principal component analysis, resolution phase peritoneal PKH2^hi^ and PKH2^lo^ macrophage datasets were normalized to the resident macrophage RNAseq dataset from Lavin et al. (7) or to ImmGen OpenSource (24) using rlog utility of DESeq2 package (25). Alternatively, the same datasets were normalized to liver macrophage microarray datasets from Zigmond et al. (19), processed with robust multi-array average (RMA) of oligo package (26) and followed by quantile normalization. Combined datasets were corrected for batch effects using ComBat utility of SVA package (27). Data analysis was performed using the R program (https://www.r-project.org/). The accession number for the RNA-seq reported in this manuscript is BioProject: PRJNA390886.

## Results

### Resolution Phase Non-phagocytic Myeloid Cells Contain Two Subsets of Ly6C^+^ Monocytic Cells in Addition to Satiated Macrophages

**Figure 1:**
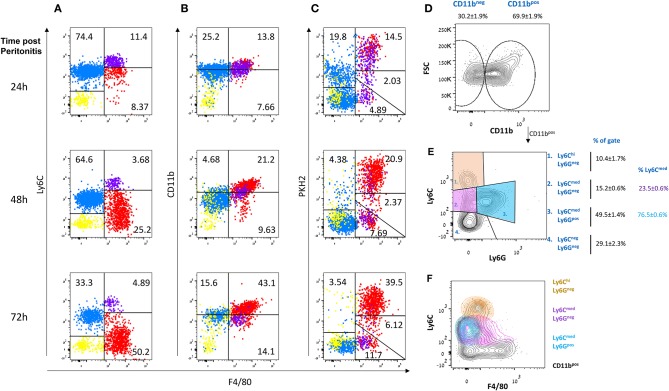
Non-phagocytic myeloid cells in peritoneal exudates contain monocytes and macrophages. Zymosan A (1 mg/mouse) was injected intraperitoneally to male mice. After 20, 44, or 68 h, these mice were injected I.P. with the phagocyte-specific dye PKH2-PCL green. Four hours later, the peritoneal cells were recovered and immunostained for F4/80 and CD11b. Dot plot analysis was performed for the expression of Ly6C (**A**, Y axis), CD11b **(B)**, and PKH2-PCL acquisition **(C)**, relative to F4/80 expression (X axis) by various exudate cells. Results are representatives from n = 8 mice for 24 h, six mice for 48 h, and seven mice for 72 h. **(D–F)** Peritoneal cells were recovered 66 h PPI and immunostained for CD11b, Ly6C, Ly6G, and F4/80 and analyzed by flow cytometry. Results are representative plots and means ± SEM (*n* = 12) showing CD11b^+^ gating **(D)**, Ly6G vs. Ly6C (identifying monocytes and neutrophils, **(E)**, and Ly6C vs. F4/80 **(F)**.

Ly6C^+^F4/80^−^ monocytes infiltrate the peritoneal cavity during the onset of resolution (12–24 h post peritonitis) and differentiate gradually to Ly6C^−^F4/80^hi^ macrophages that are highly phagocytic/efferocytic (17, 28). These phagocytic peritoneal macrophages express high levels of the macrophage surface marker CD11b, in addition to high F4/80. However, following extensive efferocytosis, they lose their phagocytic capacity and convert to a state of satiation. This phenotype conversion is accompanied by a reprogramming process and a reduction in both aforementioned surface markers (18). Recently, it was shown that non-phagocytic F4/80^+^ macrophages express high levels of IFNβ that upon secretion promotes bacterial clearance and the resolution of inflammation (17). IFNβ expression by resolution phase macrophages was also upregulated by the uptake of apoptotic cells (17). Therefore, we sought to determine whether non-phagocytic macrophages are exclusively satiated. To this end, we injected the phagocytic dye PKH2-PCL green to mice during different phases of zymosan A-induced peritonitis and analyzed the phagocytic capacity of the various myeloid phenotypes in the exudates. Our results in Figures 1A–C show that Ly6C^med^CD11b^med^F4/80^−^ monocyte-like cells are infiltrating the peritoneum at 24 h and convert at 72 h, at least in part, to Ly6C^−^CD11b^hi^F4/80^hi^ macrophages. This conversion is associated with a transition of a F4/80^−^PKH2^lo^ monocyte subset to an F4/80^+^PKH2^hi^ macrophage subset reflecting improved phagocytosis upon maturation. Unexpectedly, we observed that a significant portion of the Ly6C^+^ cells remain undifferentiated and phagocytosis reluctant even at the later phase of resolution (72 h). Also notable is the presence of a small Ly6C^hi^F4/80^lo^ subset (purple dots), commonly regarded as classical inflammatory monocytes, that is sustained during resolution but does not acquire phagocytic capacity and remains PKH2^neg^ (Figure 1C). As expected from previous reports (17, 18, 28), a subset of F4/80^hi^/PKH2^lo/neg^ cells corresponding to satiated macrophages was also evident in this analysis and distinguishable from non-phagocytic Ly6C^hi^F4/80^lo^ monocytes. Since PMN-like cells can also be part of the Ly6C^med^F4/80^neg^ population, we performed an additional analysis of CD11b^+^ cells based on Ly6C and Ly6G expression. Our results (Figures 1D–F) show that the Ly6C^med^ subset is composed of both monocytes (Ly6G^−^F4/80^lo/neg^ cells, 23.5% of Ly6C^med^) and PMN-like cells ((Ly6G^+^F4/80^neg^ cells, 76.5% of Ly6C^med^), whereas the Ly6C^hi^F4/80^lo^ subset did not contain any PMNs.

**Figure 2:**
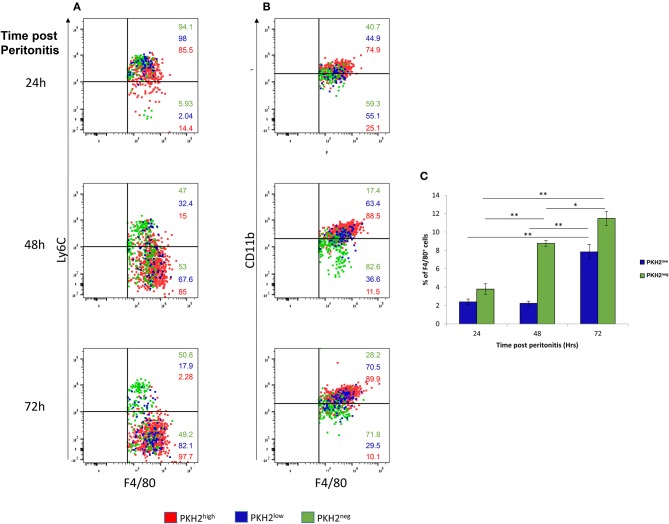
Non-phagocytic monocytes and satiated macrophages show different kinetics during the resolution of peritonitis. **(A,B)** Dot plots of F4/80^+^ PKH2-PCL high (red), low (blue), and negative (green) cells are presented relative to Ly6C **(A)** or CD11b **(B)**. **(C)** Percentage of F4/80^+^PKH2^low^ and PKH2^neg^cells at 24–72 h PPI. Results are means ± SEM (*n* = 8 mice for 24 h, six mice for 48 h, and seven mice for 72 h). *P < 0.05, **P < 0.05 (Tukey's HSD).

To better understand the phagocytic properties of F4/80^+^ myeloid subsets, we further analyzed these samples by gating on PKH2^hi^, PKH2^lo^, or PKH2^neg^ cells and analyzing their F4/80, Ly6C, and CD11b expression. Our results in Figures 2A–C show that phagocytic PKH2^hi^ cells (red dots) were initially Ly6C^+^F4/80^+^ immature monocytes, but at 72 h, they completely matured to Ly6C^−^F4/80^+^ macrophages. The satiated PKH2^lo^ macrophages (blue dots) followed a similar maturation path to the phagocytic ones, suggesting that they are indeed generated following complete maturation and loss of phagocytosis. Interestingly, the phagocytosis-reluctant F4/80^lo^ monocytes showed a very different expression of maturation markers than the other subsets. They also expressed an Ly6C^+^F4/80^+^ phenotype at 24 h, but at 72 h, only half of these cells expressed the Ly6C^−^F4/80^+^ mature phenotype. Notably, the frequency of the PKH2^neg^ cells in the exudates increased gradually with time, while the frequency of the PKH2^lo^/satiated macrophages increased only at 72 h (Figure 2C), and as previously reported, these cells contained a distinct population of CD11b^low^ macrophages (Figure 2B). Thus, non-phagocytic F4/80^+^ cells contain, in addition to satiated macrophages, phagocytosis-reluctant Ly6C^hi^F4/80^lo^monocytes.

### Ly6C^med^F4/80^neg^ and Ly6C^hi^F4/80^lo^ Cells Both Convert to Ly6C^neg^F4/80^+^ Macrophages

**Figure 3:**
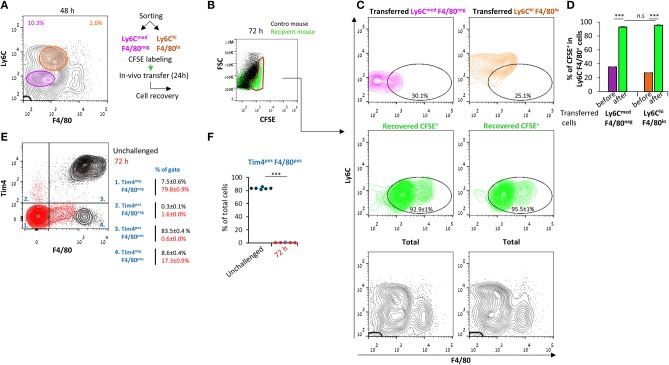
Ly6C^med^F4/80^neg^ and Ly6C^hi^F4/80^lo^ cells both convert to Ly6C^neg^ F4/80^+^ macrophages. Peritoneal exudates were recovered from WT mice 48 h PPI. **(A)** Monocytic cells were sorted into Ly6C^med^F4/80^neg^ and Ly6C^hi^F4/80^lo^ populations. **(B–D)** Sorted cells were labeled with CFSE and transferred to recipient mice with ongoing peritonitis at 48 h. At 72 h, peritoneal cells were recovered, immunostained for Ly6C and F4/80, and CFSE^+^ cells **(B)** were analyzed by flow cytometry **(C, D)**. Results are stacked contour plot from six mice **(C)** and means ± SEM (*n* = 6). *P* < 0.001 (Student's *t*-test). **(E,F)** Peritoneal exudates were recovered from unchallenged mice or at 72 h PPI, immunostained for F4/80 and Tim4 and analyzed by flow cytometry. Results are stacked contour plots from six mice **(E)** and percentage means ± SEM of F4/80^+^ Tim4^+^ cells **(F)**.***P < 0.001(Student's *t*-test).

The inflammatory monocytic subsets in the peritoneum during early resolution can differentiate to F4/80^+^ macrophages while being replaced by Ly6C^+^ cells that infiltrate from the circulation at later times. Therefore, we aimed to determine the extent of conversion of these monocytic cells to Ly6C^neg^F4/80^+^ macrophages. To this end, we sorted Ly6C^med^F4/80^neg^ (of both neutrophilic and monocytic origin) or Ly6C^hi^F4/80^lo^ cells from peritoneal exudates at 48 h PPI, labeled them with CFSE, and transferred them to the peritoneum of mice at the same phase of peritonitis. After an additional 24 h, the peritoneal cells were recovered, and the expression of maturation markers in the labeled population was examined. Our results (Figures 3A–D) show that both the Ly6C^med^F4/80^neg^ and Ly6C^hi^F4/80^lo^ subsets almost completely converted to the Ly6C^neg^F4/80^+^ phenotype. Importantly, no contribution of F4/80^hi^Tim4^+/−^ resident peritoneal cells to the F4/80^+^ macrophage subset was observed (Figures 3E,F) at this time, as previously reported for 48 h (17). Notably, the PMN-like cells were almost eliminated 24 h post transfer (Figure 3C), suggesting these cells underwent apoptosis and were engulfed by macrophages. Thus, both Ly6C^med^F4/80^neg^ and Ly6C^hi^F4/80^lo^ cells seem to be monocytes that differentiate *in vivo* to Ly6C^neg^F4/80^+^ macrophages during the resolution of inflammation.

### All Resolution Phase Monocytic/Macrophage Subsets Are CCR2 Dependent

**Figure 4:**
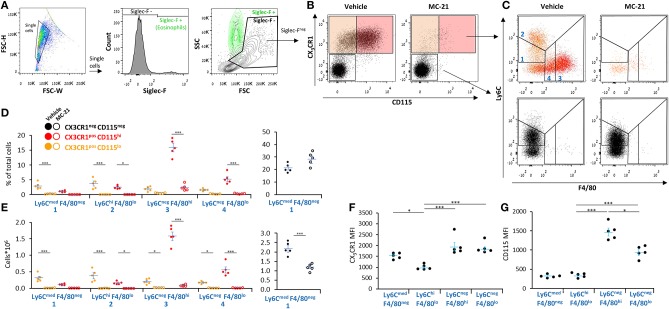
All resolution phase monocytic/macrophage subsets are CCR2-dependent. WT mice undergoing peritonitis were treated I.P. with anti-mouse CCR2 mAb (clone MC-21) or vehicle (control) at peritonitis initiation (0 h) and 24 h PPI. At 72 h, peritoneal cells were collected, immunostained for Ly6C, F4/80, CD115, CX_3_CR1, and Siglec–F and analyzed by flow cytometry. **(A)** The gating strategy excluded Siglec-F^+^ eosinophils (green). **(B–D)** Samples were analyzed according to CX_3_CR1 vs. CD115 **(B)** and CX_3_CR1^+^ (top) or CX_3_CR1^−^ (bottom) cells were analyzed according to F4/80 vs. Ly6C and CD115^hi^ (red dots) vs. CD115^lo^ subsets **(C)**. Analysis of the percentages **(D)** and cell numbers **(E)** of the indicated subsets is presented. Results are stacked dot plots **(B,C)** and means ± SEM **(D,E)** from *n* = 5. **(F,G)** CD115 **(F)** and CX_3_CR1 **(G)** expression by various CX_3_CR1^+^ myeloid subsets. Results are means ± SEM of MFI from *n* = 5. *P < 0.05, ***P < 0.001 (Student's *t*-test or Tukey's HSD).

CCR2 ligation was previously shown to be essential for monocyte recruitment and differentiation to macrophages during low-grade (0.1 mg/mouse) zymosan A-induced peritonitis (29). Therefore, we aimed to determine whether it is also essential for the recruitment of either Ly6C^med^F4/80^neg^ or Ly6C^hi^F4/80^lo^ monocytes during medium-grade peritonitis and whether its blockage during inflammation will abrogate the generation of Ly6C^−^F4/80^+^ resolution phase macrophages. To distinguish the monocytic/macrophages from PMN-like cells, we stained the cells with the monocytic markers CX_3_CR1 and CD115 (30). Our results show (Figures 4A–C) that the anti-CCR2 antibody MC-21 significantly reduced the percentages and/or peritoneal cell counts of most CX_3_CR1^+^ myeloid cells, including the Ly6C^med^F4/80^neg^CD115^lo^, Ly6C^hi^F4/80^lo^, Ly6C^neg^F4/80^hi^, and Ly6C^neg^F4/80^lo^ subsets. Notably, the percentages of the CX_3_CR1^−^Ly6C^med^F4/80^neg^PMN-like cells were not significantly changed, but their cell counts did reduce by 50%. The reduction in numbers of Ly6C^med^F4/80^neg^ PMN-like cells suggests resolution phase monocytic cells or macrophages also enhance the recruitment or delay the apoptotis/clearance of PMN-like cells during the resolution of inflammation. A comparison of the CD115 and CX_3_CR1 surface expression levels revealed similar expression of CD115 in both monocytic subsets that is increased upon maturation to macrophages and reduced following conversion to satiated Ly6C^neg^F4/80^lo^ macrophages (Figure 4H). CX_3_CR1 expression was similar on all myeloid subsets except Ly6C^hi^F4/80^lo^ monocytes that expressed significantly lower levels than all other myeloid cells (Figure 4I). Thus, our results suggest that all resolution phase monocytic cells are recruited through CCR2 or derived from CCR2-recruited precursors.

### Satiated Macrophages Are the Highest Producers of IFNβ Among Resolution Phase Leukocytes

**Figure 5:**
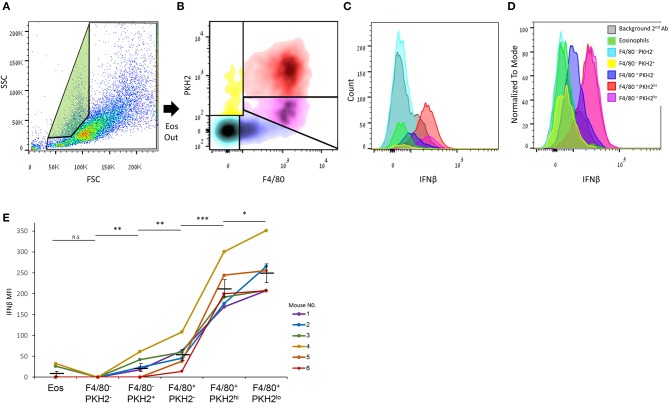
Satiated macrophages express the highest level of IFNβ of all resolution phase leukocytes. Peritoneal exudates were recovered from WT mice 66 h PPI, and the cells were immunostained for F4/80, fixed, permeabilized, and immunostained for IFNβ. **(A)** The gating strategy for eosinophils (green) and other immune cells. **(B)** Density plot analysis of F4/80 vs. PKH2 staining resulted in five distinct populations: F4/80^−^PKH2^−^ (cyan), F4/80^−^PKH2^+^ (yellow), F4/80^+^PKH2^−^ (blue), F4/80^+^PKH2^hi^ (red), and F4/80^+^PKH2^lo^ (purple). These populations were then analyzed for IFNβ expression, and results were presented as counts **(C)** and normalized to mode **(D)**. MFI means ± SEM from six independent mice are shown **(E)**. *P < 0.05, **P < 0.01, ***P < 0.001 (Student's *t*-test). Data for second antibody alone and eosinophils were previously reported in (17).

It was previously shown that non-phagocytic F4/80^+^ macrophages express higher IFNβ mRNA and protein levels in comparison with their phagocytic counterparts (17). Therefore, we aimed to determine whether this expression is exclusive to satiated F4/80^hi^PKH2^lo^ macrophages or also takes place in phagocytosis-reluctant F4/80^lo^PKH2^neg^ monocytes, or other resolution phase leukocytes. Our flow cytometry analysis (Figures 5A–D) shows that satiated F4/80^hi^PKH2^lo^ macrophages indeed express the highest amount of IFNβ of all the analyzed leukocyte subsets. F4/80^lo^PKH2^neg^ monocytes/macrophages express significantly lower levels of IFNβ than satiated macrophages, whereas eosinophils and F4/80^−^PKH2^+^ monocytes express even lower amounts of this cytokine. Surprisingly, we found phagocytic F4/80^hi^PKH2^hi^ macrophages to express high levels of IFNβ protein, but not as high as their satiated counterparts (Figure 5E). Thus, satiated macrophages seem to be the major producer of IFNβ in resolution phase exudates.

### The Transcriptome of Resolution Phase Macrophages Is More Similar to Monocytes Than to Resident Peritoneal Macrophages

**Figure 6:**
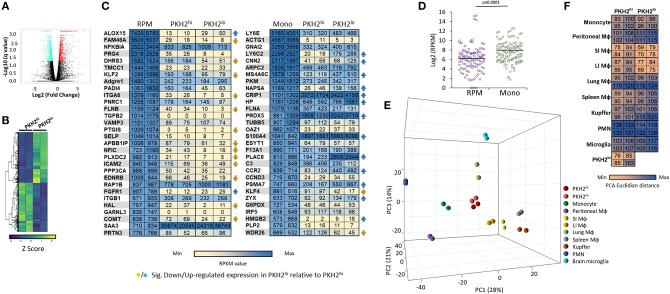
Transcriptomic analysis of PKH2^hi^/phagocytic and PKH2^lo/neg^/non-phagocytic resolution phase macrophages. Male C57BL/6 mice were injected intraperitoneally with zymosan A (1 mg/mouse) followed by an injection of PKH2-PCL at 62 h. Four hours later, the peritoneal cells were recovered and immunostained for F4/80 and CD11b. Then, F4/80^+^ macrophages were sorted based on the extent of PKH2-PCL acquisition (PKH2^hi^ vs. PKH2^low/neg^ populations; >98% purity) using the FACSAria II sorter [as reported in (17)]. The collected cells were immediately used for RNA extraction (with RNA integrity value above 7.5), and a gene expression microarray analysis was performed using Illumina hiSeq 2500. Annotated genes were plotted using a volcano plot to identify the significant differentially expressed genes comparing PKH2^hi^ and PKH2^lo^ macrophages with significance depicted at *q* ≤ 0.05 values **(A)**. Differentially expressed genes were examined across samples and hierarchically clustered into HeatMap of two lists: 1,690 up- and 1,752 down-regulated genes in PKH2^lo^ relative to PKH2^hi^. Data presented are Z score normalized **(B)**. Annotated genes were examined in comparison to various resident murine macrophage populations, as well as monocytes and PMNs [database from (7)]. The 30 highest expressed genes (on CPM-TMM scale) from either resident peritoneal macrophages (out of 282 exclusive genes) or monocytes (out of 272 exclusive genes) were compared to PKH2^hi^ and PKH2^lo/neg^ macrophages by RPKM values **(C)** and by distribution around the expression median values of each sample **(D)**. Differential distances of PKH2^hi^ and PKH2^lo/neg^ macrophages from resident peritoneal macrophages and from monocytes were visualized on a 3D PCA plot **(E)** and enumerated as PCA Euclidian distances **(F)**.

Previous studies have debated regarding the contribution of monocyte-derived inflammatory macrophages and their yolk sack-derived resident peritoneal counterparts in spontaneously resolving zymosan A-induced peritonitis (9, 17, 18, 28, 31). In order to improve our understanding of the transcriptomic origin of resolution phase macrophages and the changes that take place during the satiation process, mice were injected I.P. with PKH2-PCL at 62 h post zymosan A-induced peritonitis. After an additional 4 h, peritoneal macrophages were sorted, using flow cytometry, based on their phagocytic uptake of PKH2-PCL (17). The RNA from sorted PKH2^hi^ and PKH2^lo/neg^ macrophages was sequenced, and a total of 31,727 genes were annotated. A volcano plot was generated from the obtained data in order to assess the 3,442 differentially expressed genes (*q* ≤ 0.05, 10.9% of annotated genes) (Figure 6A). The produced differential gene list is presented across all the samples as a HeatMap (Figure 6B), in which hierarchical clustering generated two lists: 1,690 upregulated genes and 1,752 downregulated genes in PKH2^lo^ relative to PKH2^hi^ macrophages.

We previously indicated that select genes from RPM are barely expressed in either phagocytic or non-phagocytic resolution phase macrophages from zymosan A-induced peritonitis, while monocyte markers are abundantly expressed in these cells (17). Since macrophages are able to change their transcriptome in an environment-specific manner (7), we aimed to further characterize the transcriptomes of phagocytic and satiated macrophages to determine whether genes expressed by peritoneal macrophages are also substantial in resolution phase macrophages. To this end, we designated 30 genes with the highest specific expression in either RPM or monocytes based on Lavin et al. (7) and compared their expression to phagocytic and non-phagocytic macrophages. Our results (Figure 6C) indicate that some genes (i.e., rap1b, saa3, and nfkbia) highly expressed by RPM are also abundantly expressed by phagocytic and non-phagocytic resolution phase macrophages. However, neither phagocytic nor non-phagocytic macrophages expressed notable mRNA levels of markers of RPM, such as *timd4* [3.47 and 1.26 reads per kilobase million (RPKM) for phagocytic and non-phagocytic macrophages, respectively], *vsig4* (2.58 and 4.39 RPKM, respectively), *nt5e* (1.31 and 2.24 RPKM, respectively), and *cd209b* (0 and 0.22 RPKM, respectively). Unexpectedly, although the canonical RPM transcription factor GATA6 is not expressed in resolution phase macrophages (17), some genes regulated by this transcription factor (8, 9), such as cd9 (139.19 and 266.25 RPKM for phagocytic and satiated macrophages, respectively), cd24a (68.35 and 1198.62 RPKM, respectively), and cd93 (44.35 and 12.37, respectively) were expressed by both phagocytic and non-phagocytic resolution phase macrophages and their levels were significantly modulated upon phenotype conversion.

Overall, analysis of our transcriptomic data against the 30^th^ highest expressed genes in RPM and monocytes indicated a significantly increased median RPKM value for resolution phase macrophages of both phenotypes toward monocyte genes (Figure 6D) than toward their RPM counterparts. Moreover, analysis of the percentage of genes that were expressed at 10 RPKM or lower levels revealed a significantly higher percentage in RPM than in monocyte genes (Table 1). Principal component analysis (PCA) and calculation of PCA Euclidian distances revealed that PKH2^hi^ (phagocytic) and PKH2^lo^ (non-phagocytic) macrophages are positioned closer to one another, as well as to the small intestine and large intestine macrophages than to any other myeloid subset presented (Figures 6E,F). Moreover, both phagocytic and satiated resolution phase macrophages were positioned closer to monocytes than to RPM (Figures 6E,F). Of interest, intestinal macrophages that have many common features with monocytes (7) were the closest resident macrophage subset to resolution phase macrophages (Figures 6E,F). Notably, non-phagocytic macrophages were found to increase the expression of monocyte genes, in comparison to phagocytic macrophages (14 of 15 genes that were modulated in a statistically significant manner), whereas the expression of RPM genes was decreased in these cells (15 of 21 genes) (Figure 6C). Thus, our transcriptomic analysis indicates that both phagocytic and non-phagocytic resolution phase macrophages are monocyte-derived with similarities to resident peritoneal and intestinal macrophages.

**Table 1:** RPM and monocyte genes under-represented in resolution phase macrophages.

	**PKH2^**hi**^ (%)**	**PKH2^**lo**^ (%)**
RPM genes <10 RPKM	8.33	21.67
Monocyte genes <10 RPKM	1.67	3.33

*The percentage of genes from phagocytic (PKH2^hi^) and (PKH2^lo^) macrophages that were expressed at lower than 10 RPKM values in the 30^th^ highest expressed genes in RPM and monocytes*.

### Resolution Phase Macrophages Resemble Liver Reparative Ly6C^lo^ Macrophages and Peritoneal Monocyte-Derived Macrophages Elicited by Thioglycolate

**Figure 7:**
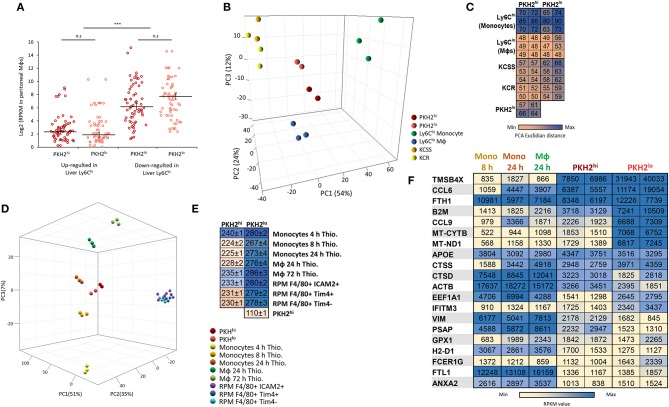
Resolution phase macrophages resemble liver reparative Ly6C^lo^ macrophages and peritoneal monocyte-derived macrophages elicited by thioglycolate. Annotated genes were compared to the database of monocyte/macrophage populations from acute liver injury induced by overdose of N-acetyl-p-aminophenol (APAP) [Zigmond et al. (19)]. These subsets include inflammatory Ly6C^hi^ monocytes and their descendants, Ly6C^lo^ monocytes, as well as Kupffer cells from the steady state (KCSS) and recovered (KCR) phases. The 50 highest up- or downregulated genes in the liver Ly6C^lo^ differentiated macrophages were compared to PKH2^hi^/phagocytic and PKH2^lo^/^neg^/non phagocytic macrophages **(A)**. Differential distances of PKH2^hi^ and PKH2^lo/neg^ macrophages from liver macrophages and monocytes were visualized on a 3D PCA plot **(B)** and enumerated as PCA Euclidian distances **(C)**. Alternatively, annotated genes were compared to the database of resident tissue macrophages and thioglycolate-elicited peritoneal monocyte/macrophage populations from the ImmGEN consortium (OpenSource mononuclear phagocyte project). The peritoneal resident populations were designated as RPM F4/80^+^ICAM2^+^ (F4/80^+^ICAM2^+^CD3^−^CD19^−^Ter119^−^) and RPM F4/80^+^ Tim4^+^/Tim4^−^ (B220^−^Ly6C^−^F480^+^CD11b^+^CD64^+^Tim4^+^/Tim4^−^). The peritoneal thioglycolate-elicited populations were designated as follows: monocytes 4 and 8 h Thio (CD45^+^CD11b^+^CD115^+^Ly-6C^+^ICAM2^−^CD226^−^), monocytes 24 h Thio (CD45^+^CD11b^+^CD115^+^Ly-6C^+^CD36^lo^ICAM2^−^CD226^−^), and macrophages 24 and 72 h Thio (CD45^+^CD11b^+^CD115^+^Ly-6C^lo^CD36^+^ICAM2^−^CD226^−^). Differential distances of PKH2^hi^ and PKH2^lo/neg^ macrophages from peritoneal resident, and thioglycolate-elicited monocytes/macrophages were visualized on a 3D PCA plot **(D)** and enumerated as PCA Euclidian distances presented as group to group mean ± SEM **(E)**. The 20 highest expressed genes (on CPM-TMM scale) from either PKH2^hi^ or PKH2^lo/neg^ macrophages were compared to monocytes 4 and 8 h Thio and macrophages 24 h Thio by RPKM values **(F)**.

Acetominophen-induced liver injury, like zymosan A-induced peritonitis, is hallmarked by inflammatory Ly6C^hi^ monocyte differentiation to reparative Ly6C^lo^ macrophages and the clearance of apoptotic neutrophils (19–21). Therefore, we compared the transcriptome of these liver-associated, monocyte-derived cells to peritoneal phagocytic and non-phagocytic macrophages. Our results show that in the 50 highest-fold changed genes downregulated in liver Ly6C^lo^ macrophages, there is a significantly higher expression in both peritoneal phagocytic and non-phagocytic macrophages, compared to the upregulated genes (Figure 7A). Interestingly, the PCA and Euclidian distance analysis revealed an increased similarity of both phagocytic and non-phagocytic macrophages to Ly6C^lo^ macrophages rather than to their Ly6C^hi^ precursors or Kuppfer cells (Figures 7B,C), thus suggesting that the 50 highest expressed genes are less indicative of transcriptomic changes in this analysis. In addition, comparison of resolution phase macrophages and thioglycolate-elicited monocytes/macrophages analyzed by the ImmGEN consortium (24) revealed that both phagocytic and non-phagocytic macrophages show the highest resemblance to monocytes and macrophages elicited at 8–24 h post thioglycolate administration (PTA). These macrophages showed lower similarity to monocytes or macrophages recovered at 4 or 72 h PTA, respectively, or to various subsets of resident peritoneal macrophages (Figures 7D–F). Notably, phagocytic and non-phagocytic macrophages showed a significantly higher resemblance to one another (two fold) than to any other monocyte/macrophage subset, thus, underscoring their common origin. Together, this analysis suggests that the transcriptomic profile of both phagocytic and non-phagocytic macrophages resembles reparative macrophages from liver injury, and peritoneal monocyte-derived macrophages, which might contain or mature into both subsets. These results also suggest that the Ly6C^hi^F4/80^lo^ monocytic subset does not contribute significantly to the transcriptome of non-phagocytic macrophages that is rather dominated by satiated macrophages.

### Transcriptomic Modulation in Non-phagocytic/Satiated Macrophages Supports a Role in Limiting Tissue Repair and Fibrosis

**Figure 8:**
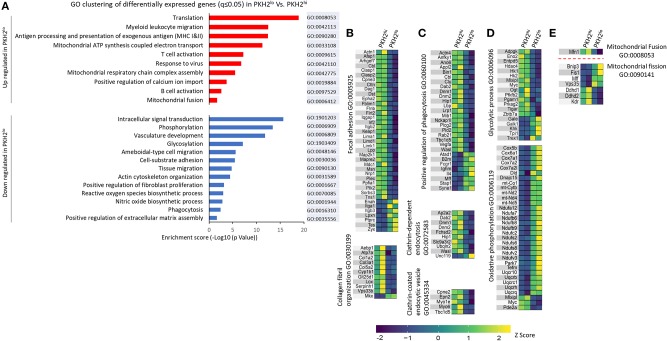
Select functional GO pathways skewed in non-phagocytic macrophages. Analysis of gene enrichment for biological processes and KEGG pathway was performed on the differential up- and down-regulated gene clusters **(A)**. A search for GO pathways was performed to examine skewed functions comparing PKH2^hi^ and PKH2^lo^ macrophages in terms of tissue repair and fibrosis **(B)**, phagocytic activity **(C)**, bioenergetics **(D)**, and mitochondrial dynamics **(E)**. Data presented are differentially expressed (twofold change) genes from each GO term category with *q* ≤ 0.05.

In order to analyze the nature of the differential gene clustering and the potential variation in the properties of non-phagocytic macrophages, both upregulated and downregulated gene lists were separately analyzed by GO enrichment for biological processes and KEGG pathways at DAVID Bioinformatics Resources 6.7, National Institute of Allergy and Infectious Diseases (NIAID), NIH. Enrichment output was clustered into 88 upregulated and 143 downregulated biological processes together with three upregulated and 10 downregulated KEGG pathways. The 23 select clusters from the upregulated and downregulated genes (Figure 8A) represent fundamental shifts in cell metabolism, phagocytic activity, tissue interaction and repair, and paracrine modulation of inflammatory processes progress. Based on the above and in order to better understand the genes involved in macrophage phenotype acquisition in terms of modulation of phagocytosis, tissue repair, metabolism, and immune activity, a supervised search toward GO pathways was conducted based on MGI (32). Our results in Figure 8 show several phenotypic shifts at the transcriptomic level that are associated with macrophage loss of phagocytosis. Satiated macrophages show a significant reduction in the expression of gene clusters involved in intracellular signal transduction, vascular development, cell–substrate adhesion, actin cytoskeleton organization, and positive regulation of both fibroblast proliferation and extracellular matrix organization. These changes suggest a shift from an M2-like/reparative phenotype to a pro-resolving phenotype. Moreover, satiated macrophages express reduction in gene clusters involving collagen organization and focal adhesion (Figure 8B). These are two important gene clusters for macrophages that mediate tissue repair and wound healing, but also tissue fibrosis and scarring that leads to organ failure (33). Bitterman and colleagues previously indicated that fibrotic ECM can initiate a pro-fibrotic cycle in fibroblasts that leads to idiopathic pulmonary fibrosis (IPF) (34). Notably, of the 28 genes that were both significantly changed in IPF patients and significantly downregulated in satiated macrophages (two fold), 26 were upregulated, while two were downregulated in IPF patients (Table 2). These findings support the notion that resolution phase macrophages deviate from their M2/pro-fibrotic phenotype upon conversion from phagocytic to satiated macrophages and that M2-like resolution phase macrophages might promote tissue fibrosis by directly producing ECM components in addition to regulating fibroblast proliferation and ECM deposition.

**Table 2:** Comparative analysis of genes modulated in satiated macrophages and IPF patients.

**Description**	**Symbol**	**Fold reduction (–log_**2**_)**
Laminin B1	Lamb1	5.73342
Collagen, type I, alpha 2	Col1a2	3.72915
Collagen, type Ill, alpha	Col3a1	3.65418
Secreted acidic cysteine rich glycoprotein	Sparc	3.26993
Lysyl oxidase-like 1	Loxl1	3.17508
Collagen, type V, alpha 2	Col5a2	3.16363
Cysteine rich protein 61	Cyr61	2.999
Bone morphogenetic protein 1	Bmp1	2.37722
Serine (or cysteine) peptidase, inhibitor, clade G, member 1	Serping1	2.85737
Collagen, type VI, alpha 2	Col6a2	2.60818
Laminin, beta 2	Lamb2	2.51054
Syntaxin 1A (brain)	Stx1a	2.36814
Follistatin-like 1	Fstl1	2.35297
Vasorin	Vasn	2.27123
Collagen, type VI, alpha 1	Col6a1	2.23102
Cysteine rich transmembrane BMP regulator 1 (chordin like)	Crim1	2.22188
Lysyl oxidase-like 3	Loxl3	1.92259
Niemann Pick type C1	Npc1	1.75604
Collagen, type I, alpha 1	Col1a1	1.63809
Scavenger receptor cysteine rich domain containing (5 domains)	Ssc5d	1.53205
platelet-derived growth factor C polypeptide	Pdgfc	1.48094
Tissue inhibitor of metalloproteinase 2	Timp2	1.38781
Phospholipid transfer protein	Pltp	1.29273
Latent transforming growth factor beta binding protein 3	Ltpb3	1.24257
C-type lectin domain family 11, member a	Clec11a	1.17277
Filamin, alpha	Flna	1.16859
Elastin microfibril interfacer 2	Emilin2	1.13144
Laminin, gamma 1	Lamc1	1.10757

*A list of all the genes that were significantly down-regulated in satiated macrophages and significantly changed in samples for IPF patients. Genes labeled in yellow were up-regulated while genes labeled in gray were down-regulated in IPF patients*.

Previous reports have indicated that satiated macrophages lose their phagocytic potential upon conversion from their phagocytic counterparts and migrate to remote sites (17, 18). Our results in Figure 8A show that satiated macrophages downregulate gene clusters, such as phagocytosis, actin cytoskeleton organization, and ameboidal-type cell migration, while increasing clusters like myeloid leukocyte migration. Moreover, our GO analysis indicates that satiated macrophages mostly downregulate positive regulation of phagocytosis, clathrin-dependent endocytosis, and clathrin-coated endocytic vesicles (Figure 8C). These findings support the notion that phagocytic macrophages undergo a process of satiation that results in a loss of their phagocytic properties and their controlled departure of the injury site during the resolution of inflammation.

Previous studies in the last 20 years have indicated that a broad metabolic switch takes place during macrophage differentiation to M1- and M2-like phenotypes. While bacterial and inflammatory stimuli induce glycolytic pathways in macrophages that acquire M1-like features, oxidative phosphorylation and the TCA cycle are the preferred metabolic processes in M2-like macrophages (35). Our results reveal a similar dichotomy in phagocytic and satiated macrophages during the resolution of inflammation. Figure 8D shows increased expression of genes involved in mitochondrial ATP synthesis-coupled electron transport and respiratory chain complex assembly that compose an oxidative phosphorylation cluster, while genes included in the glycolytic process are downregulated. Moreover, genes associated with NO biosynthesis, a hallmark of M1 macrophages, are downregulated in satiated macrophages (Figure 8A). Notably, additional mitochondrial processes seem to take place on the transcriptomic level during satiation. Only one gene, mitofusin-1 (Mfn1), is significantly downregulated in the mitochondrial fusion cluster. However, this is a key regulator of mitochondria fusion (36). On the other hand, four genes associated with mitochondrial fission were upregulated in satiated macrophages (Figure 8E). Unexpectedly, the other three genes involved in mitochondrial fission were downregulated in satiated macrophages. However, these genes are also involved in other processes that are downregulated in these macrophages, such as inhibition of oxidative phosphorylation and blood vessel morphogenesis. ROS production is also downregulated in satiated macrophages by reducing the expression of this gene cluster specifically (Figure 8A). Thus, satiated macrophages seem to regulate the expression of various gene clusters involved in important functions that these cells execute highlighted by limiting excessive tissue repair and fibrosis.

## Discussion

The emergence of satiated Ly6C^−^F4/80^+^CD11b^low^ macrophages that contained high numbers of apoptotic cell nuclei but engulfed low levels of the phagocytosis-acquired dye PKH2-PCL *in vivo* was previously reported during the resolution phase of murine peritonitis (17, 18). These macrophages were converted from phagocytic Ly6C^−^F4/80^+^CD11b^high^ that contained low numbers of apoptotic cell nuclei. The expression and secretion of IFNβ by non-phagocytic F4/80^+^ macrophages was recently reported (17), and therefore, it was of interest to determine whether satiated macrophages are the only non-phagocytic myeloid subset. Surprisingly, our results revealed, in addition to the satiated F4/80^+^PKH2^lo^ macrophage subset, two other subsets of Ly6C^+^ monocytes in resolving exudates. One subset was characterized as Ly6C^med^F4/80^neg^ monocytes that initially displayed low phagocytic capacity (at 24 h PPI). However, at 72 h PPI, the low phagocytic monocytes seem to differentiate to Ly6C^−^F4/80^+^ macrophages with high phagocytic capacity. Notably, a significant portion of these monocytes do not become mature and phagocytic even at 72 h PPI, suggesting that these phagocytosis-reluctant monocytes are key regulators of the resolution of inflammation on site. The second population of non-phagocytic monocytes is characterized as Ly6C^hi^F4/80^lo^monocytes. The frequency of these F4/80^lo^PKH2^neg^ cells is increasing continuously during the transition from the inflammatory to the resolving phases of peritonitis (Figure 2C) without acquiring any phagocytic activity. At 72 h PPI, only 50% of these cells are Ly6C^−^F4/80^+^, while transfer experiments showed that almost all of these cells become Ly6C^−^F4/80^+^ within 24 h of peritoneal maturation. Thus, this non-phagocytic Ly6C^hi^F4/80^lo^CXC_3_CR1^+^CD115^lo^ population also seems to be supplemented by blood-borne precursors, while maturing *in vivo* to an Ly6C^−^F4/80^+^ phenotype without acquiring phagocytic capacity. Importantly, these PKH2^neg^ monocytes contain a higher percentage of CD11b^low^ cells than their PKH2^lo^ satiated counterparts (18) (Figure 2B) at 48 and 72 h, suggesting that modulation of CD11b expression is important for both acquisition and loss of phagocytosis capacity. It is important to note that the aforementioned changes in macrophage phenotypes should take into account the migration of young monocytes to the peritoneum that replenishes the non-phagocytic populations and the emigration of mature macrophage to remote sites that diminishes the frequency of phagocytic and/or satiated macrophages.

Since the expression of IFNβ by non-phagocytic macrophages was performed using a gating strategy that did not discriminate F4/80^+^ satiated and phagocytosis-reluctant monocytes, we used flow cytometry to directly evaluate IFNβ expression by each resolution phase leukocyte subset. We found (Figure 5) that F4/80^−^PKH2^+^ and F4/80^lo^PKH2^neg^ monocytes expressed very low amounts of IFNβ. Phagocytic (F4/80^hi^PKH2^hi^) and satiated (F4/80^hi^PKH2^lo^) macrophages, however, expressed high levels of this cytokine with the latter being significantly superior to all other leukocyte subsets. Notably, while phagocytic macrophages were found to express low levels of IFNβ mRNA and non-secreted isoforms of this protein, they did express higher levels of the secreted isoform (17), which could explain the relatively high detection of IFNβ protein by flow cytometry.

The big disparity in IFNβ expression between satiated and phagocytosis-reluctant monocytes suggests that the former are the major contributors to the transcriptome of non-phagocytic macrophages, especially considering the many IFN-responsive genes upregulated in non-phagocytic macrophages (17). These findings are also supported by the lack of difference in F4/80 expression between phagocytic and non-phagocytic macrophages (237.6 and 239.5 RPKM, respectively), whereas flow cytometry shows a twofold difference between Ly6C^hi^F4/80^lo^ and Ly6C^−^F4/80^hi^ cells (data not shown, *N* = 6). In addition, the relative similarity of the transcriptomes of both resolution phase macrophage subsets and resolution phase reparative Ly6C^lo^ macrophages from liver injury, compared to their Ly6C^hi^ monocyte counterparts, underscores the contribution of mature satiated macrophages rather than immature monocytes to the transcriptome of non-phagocytic macrophages. Notably, both resolution phase macrophage subsets had a higher transcriptomic similarity to monocytes and monocyte-derived macrophages rather than RPM. These findings are in accord with previously published results (17) and further support the notion that resolution phase macrophages in this zymosan A-induced inflammation are monocyte-derived. Non-phagocytic macrophages showed some increased transcriptomic similarity to monocytes than their phagocytic counterparts (Figure 6C). Therefore, we cannot exclude some contribution of Ly6C^hi^F4/80^lo^ cells to their transcriptome. Nevertheless, it seems that the transcriptome of non-phagocytic macrophages is dominated by the satiated subset, and we will further discuss the function of these cells as satiated macrophages.

A comparison of the transcriptomes of phagocytic and satiated macrophages suggest that satiation is associated with an M1-to-M2 metabolic shift, namely, from glycolysis to oxidative phosphorylation, that is maintained during the resolution sequel, while satiated macrophages transition from a pro-fibrotic phenotype to a pro-resolving one. The increase in mitochondria fission and the reduction in ROS biosynthetic clusters seems linked to the high oxidative burden (from apoptotic debris) (15) that satiated macrophages need to tolerate, possibly by reducing their production of ROS. Thus, satiated macrophages seem to adjust to the balance between loss of the phagocytic machinery and the need to degrade cellular constituents and control ROS production.

Notably, we found satiated macrophages to upregulate gene clusters associated with T- and B-cell activation as well as responses to viruses. The unique IFNβ-associated gene signature previously observed in these macrophages (17) and the role of some inflammatory cytokines and chemokines in the resolution phase of inflammation (37–40) can partially account for this gene regulation. However, it is also documented that inflammatory cytokines, like TNFα, play a role in limiting muscle fibrosis by promoting the death of fibro/adipogenic progenitors in affected tissues (41). Resolution phase macrophages can also play a significant role in bridging the gap between innate and acquired immunity by attracting various myeloid subsets to the resolving site and affecting lymphoid responses (29).

In conclusion, we have shown that the resolution of inflammation yields several species of phagocytosis-reluctant and satiated myeloid cells, as well as phagocytic macrophages. The comparative analysis of the transcriptomes of satiated macrophages and their phagocytic precursors reveals a distinct shift in gene clusters that correspond to phagocytic, metabolic, and inflammatory properties. These genes and pathways are highlighted in the current report, suggesting a tissue repair and fibrosis-limiting role for satiated macrophages, and serving as a prelude to further studies that will decipher the intricate properties of resolution phase macrophages in various organs and inflammatory models.

## Data Availability Statement

The datasets analyzed for this study can be found in the BioProject repository with accession number PRJNA450293.

## Ethics Statement

The animal study was reviewed and approved by the committee of Ethics in Animal Experimentation, University of Haifa.

## Author Contributions

SB and SS isolated macrophages extracted RNA and performed bioinformatics analysis of the sequences obtained. SB also performed the transfer, monocytic ablation, and MDSC characterization experiments, and wrote the manuscript. SS and SA performed the myeloid cell characterization. SS-Z and NS assisted in RNA isolation and data analysis. DB assisted in data analysis and discussion. AA designed the study and wrote the manuscript.

### Conflict of Interest

The authors declare that the research was conducted in the absence of any commercial or financial relationships that could be construed as a potential conflict of interest.

## Supplementary Material

Supplementary Figure 1 Gating strategy for analysis of monocyte/macrophage subsets. Immunostained peritoneal cells from 24–72 h PPI were plotted as FSC vs. SSC, and small apoptotic cells and lymphocytes, as well as granulocytic eosinophils were excluded from the analysis **(A)**. Then, single cells were gated for further analysis **(B)** according to Ly6C vs. F4/80 **(C)**, CD11b vs. F4/80 **(D)**, and PKH2 vs. F4/80 **(E)**.
