# Corrigendum: Transcriptomic Analysis of Monocyte-Derived Non-Phagocytic Macrophages Favors a Role in Limiting Tissue Repair and Fibrosis

**DOI:** 10.3389/fimmu.2020.01003

**Published:** 2020-05-20

**Authors:** Sergei Butenko, Senthil K. Satyanarayanan, Simaan Assi, Sagie Schif-Zuck, Dalit Barkan, Noa Sher, Amiram Ariel

**Affiliations:** ^1^Department of Human Biology, University of Haifa, Haifa, Israel; ^2^Tauber bioinformatics Center, University of Haifa, Haifa, Israel

**Keywords:** inflammation, macrophages, efferocytosis, transcriptional profiling, fibrosis

Following a reassessment of the contribution of Dr. Dalit Barkan to discussions on the database presented in the manuscript, the authors came to the conclusion that her name should be included in the author list.

## Author Contributions

SB and SS isolated macrophages extracted RNA and performed bioinformatics analysis of the sequences obtained. SB also performed the transfer, monocytic ablation, and MDSC characterization experiments, and wrote the manuscript. SS and SA performed the myeloid cell characterization. SS-Z and NS assisted in RNA isolation and data analysis. DB assisted in data analysis and discussion. AA designed the study and wrote the manuscript.

The authors apologize for this error and state that this does not change the scientific conclusions of the article in any way. The original article has been updated.

