# Supplementary figures and images for: Transcriptomic Analysis of Monocyte-Derived Non-Phagocytic Macrophages Favors a Role in Limiting Tissue Repair and Fibrosis
Source: Front Immunol. 2020 Mar 31;11:405. doi: 10.3389/fimmu.2020.00405 (PMC7136412; doi:10.3389/fimmu.2020.00405)

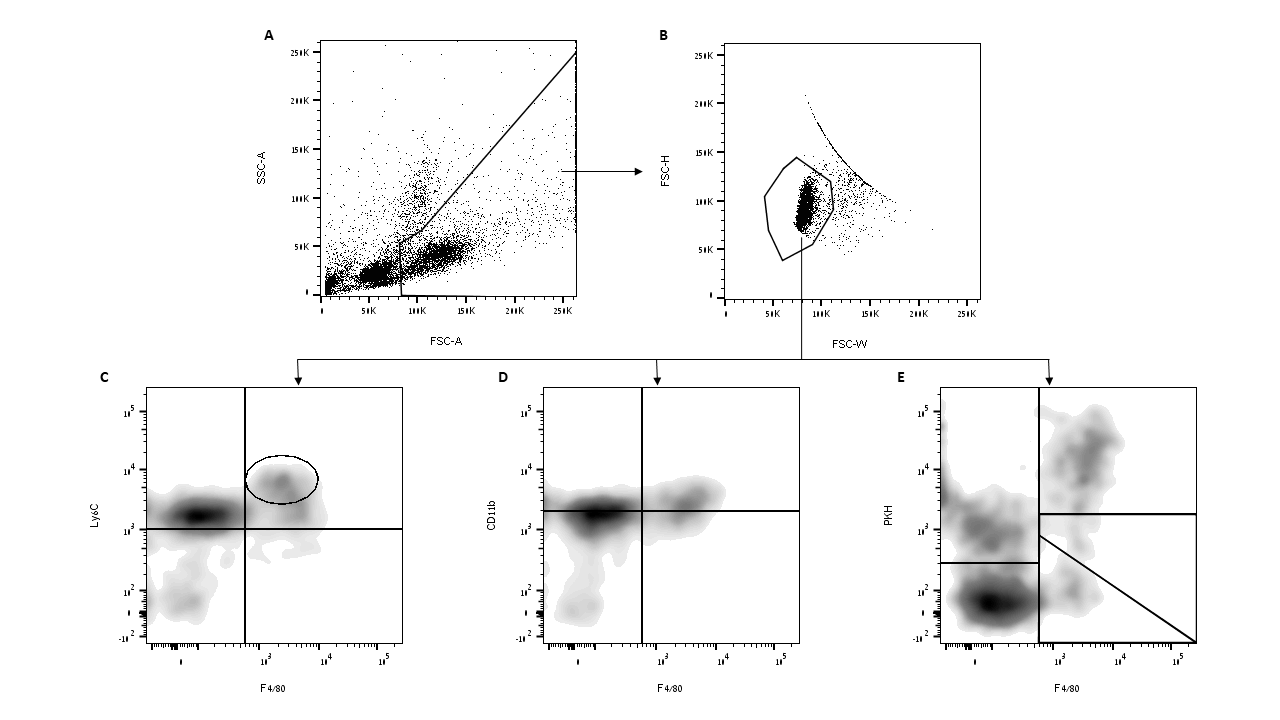

Supplement: Supplementary Figure 1 — Gating strategy for analysis of monocyte/macrophage subsets. Immunostained peritoneal cells from 24–72 h PPI were plotted as FSC vs. SSC, and small apoptotic cells and lymphocytes, as well as granulocytic eosinophils were excluded from the analysis (A). Then, single cells were gated for further analysis (B) according to Ly6C vs. F4/80 (C), CD11b vs. F4/80 (D), and PKH2 vs. F4/80 (E). [file Image_1.TIF]
